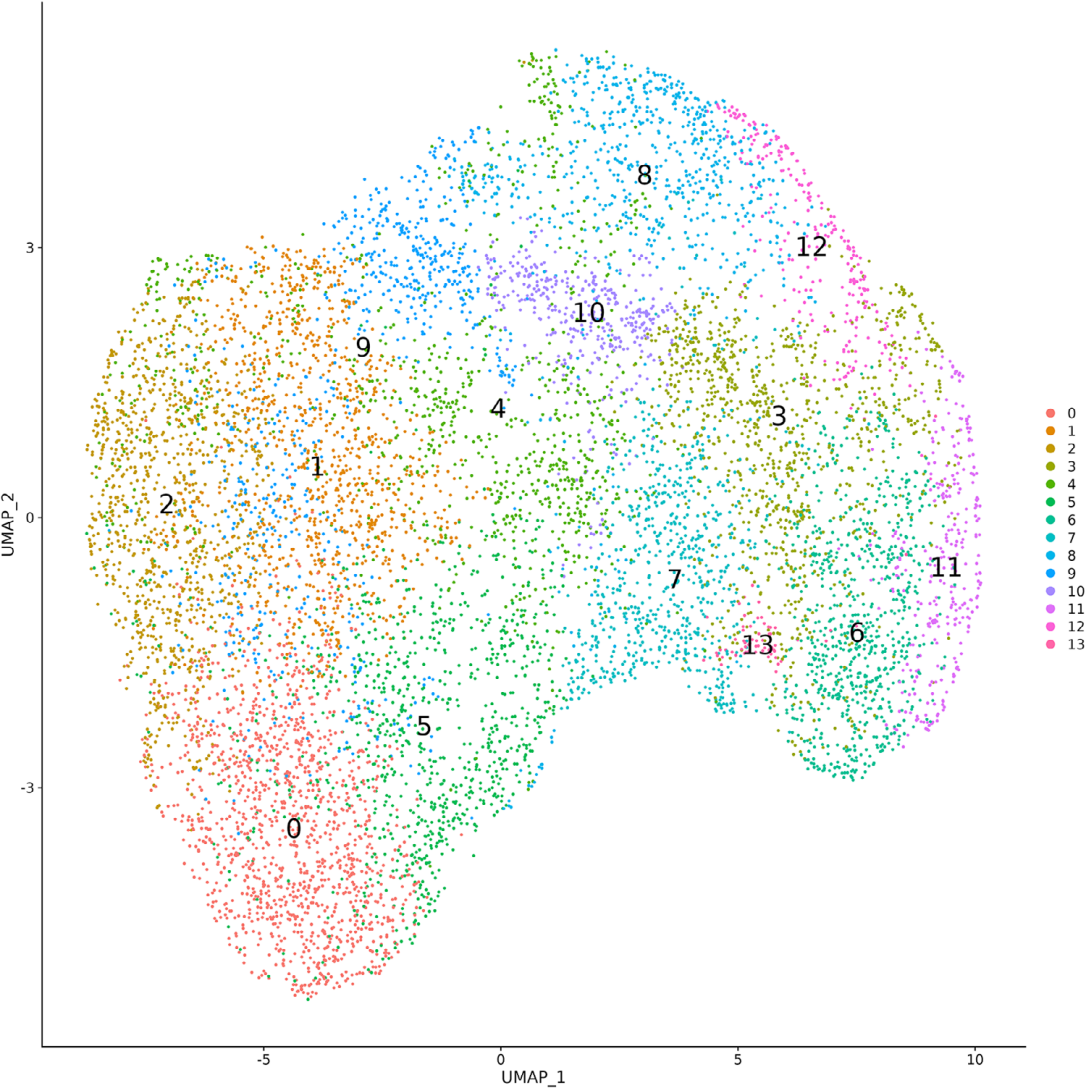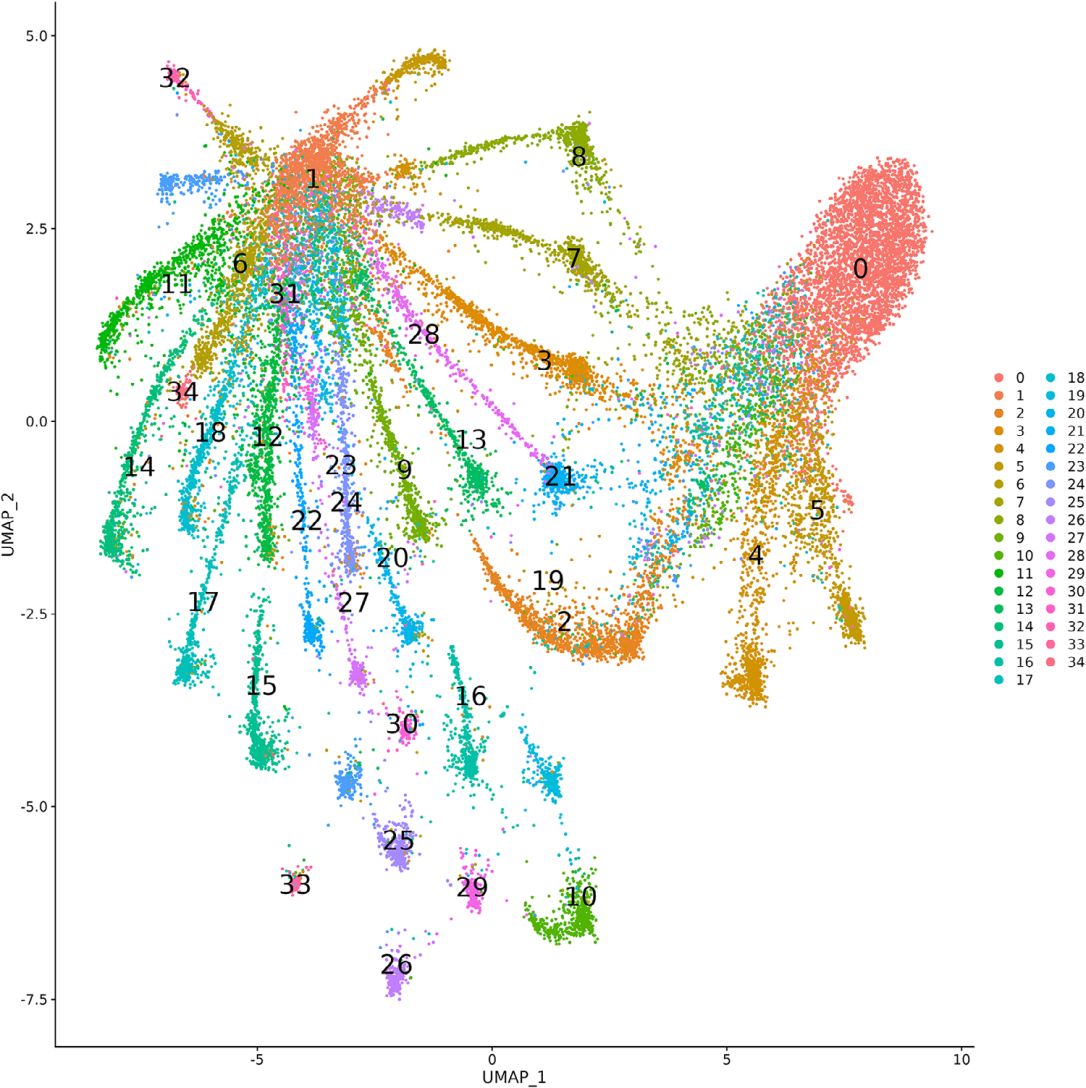# Single‐cell spatial and non‐spatial RNA sequencing on herpes simplex virus 1 (HSV‐1) infected 2D and 3D cerebral organoids reveal molecular granularity on virus‐human gene interactions in Alzheimer’s disease

**DOI:** 10.1002/alz.092866

**Published:** 2025-01-03

**Authors:** Pepper Dawes, Nathaniel J Barton, Adrian R Orszulak, Samantha M Chigas, Khanh Tran, Jonathan Sundstrom, Meagan N Olson, Liam F Murray, HyungSuk Oh, George M Church, David M Knipe, Benjamin Readhead, Yingleong Chan, Elaine T Lim

**Affiliations:** ^1^ UMass Chan Medical School, Worcester, MA USA; ^2^ Harvard Medical School, Boston, MA USA; ^3^ Arizona State University, Tempe, AZ USA

## Abstract

**Background:**

Herpes simplex virus (HSV‐1) has been associated with molecular and cellular signatures associated with Alzheimer’s disease (AD). We explored the use of both recent single‐cell and bulk transcriptomics technologies in dissecting the molecular and cellular virus‐human interactions with HSV‐1 infected cerebral organoids (2D and 3D). We compared the results with our previous observations from bulk RNA sequencing and discovered novel insights into HSV‐1 induced AD‐associated molecular pathology that were made possible by each transcriptomics technology.

**Method:**

We used recent emerging technologies such as single‐cell spatial RNA sequencing (STOmics Stereo‐seq) and single‐cell non‐spatial RNA sequencing (Parse Evercode). In addition, we have data generated from our previous work using bulk RNA sequencing.

**Result:**

Previously, we found that differentially expressed genes in HSV‐1 infected dissociated cells from cerebral organoids (2D cOrgs) using bulk RNA sequencing data were exclusively enriched for AD‐associated genes implicated through genome‐wide association studies (GWAS), but not for genes associated with other neurodegenerative or autoimmune diseases.

UMAP analyses on single‐cell non‐spatial RNA sequencing data with GFP‐tagged HSV‐1 infected 2D cOrgs clustered by viral transcripts [Fig. 1] revealed huge differences in the proportions of true late viral transcripts but not of leaky late viral transcripts between clusters, while similar clustering of 3D cOrgs [Fig. 2] showed distinct proportions of both true late and leaky late transcripts.

Pseudobulk analyses showed no enrichment for AD‐associated GWAS genes among the differentially expressed genes for HSV‐1 infected 2D cOrgs versus uninfected 2D cOrgs, but revealed significant enrichment for GWAS genes associated with autoimmune diseases such as Type 1 diabetes and multiple sclerosis. Similar analyses of HSV‐1 infected 3D cOrgs versus uninfected 3D cOrgs did not show enrichment in GWAS gene lists across the 21 common neurodegenerative, neuropsychiatric or autoimmune diseases that we had surveyed.

**Conclusion:**

Taken together, our use of multi‐transcriptomics technologies has enabled us to gain course to fine molecular granularity into neuroinflammation‐induced AD viral‐human gene interactions and a careful dissection of the multi‐transcriptomics data enables us to gain insights into biomarkers and gene targets for AD therapeutics development in the near future.